# Evolution of the Degenerated Y-Chromosome of the Swamp Guppy, *Micropoecilia picta*

**DOI:** 10.3390/cells11071118

**Published:** 2022-03-25

**Authors:** Indrajit Nanda, Susanne Schories, Ivan Simeonov, Mateus Contar Adolfi, Kang Du, Claus Steinlein, Manfred Alsheimer, Thomas Haaf, Manfred Schartl

**Affiliations:** 1 Department of Human Genetics, University of Würzburg Biocenter, 97074 Würzburg, Germany; nanda@biozentrum.uni-wuerzburg.de (I.N.); claus.steinlein@biozentrum.uni-wuerzburg.de (C.S.); thomas.haaf@uni-wuerzburg.de (T.H.); 2 Developmental Biochemistry, University of Würzburg Biocenter, 97074 Würzburg, Germany; susanne.schories@biozentrum.uni-wuerzburg.de (S.S.); ivan.simeonov@uni-wuerzburg.de (I.S.); mateus.adolfi@biozentrum.uni-wuerzburg.de (M.C.A.); 3 Xiphophorus Genetic Stock Center, Texas State University, San Marcos, TX 78666, USA; dukang1117@outlook.com; 4 Department of Cell and Developmental Biology, University of Würzburg Biocenter, 97074 Würzburg, Germany; alsheimer@biozentrum.uni-wuerzburg.de

**Keywords:** sex chromosomes, heterochromatin, Y chromosome degeneration, meiosis, synaptonemal complex, recombination, 5-methylcytosine, testosterone, sexual antagonistic genes, sex linked pigmentation pattern

## Abstract

The conspicuous colour sexual dimorphism of guppies has made them paradigmatic study objects for sex-linked traits and sex chromosome evolution. Both the X- and Y-chromosomes of the common guppy (*Poecilia reticulata*) are genetically active and homomorphic, with a large homologous part and a small sex specific region. This feature is considered to emulate the initial stage of sex chromosome evolution. A similar situation has been documented in the related Endler’s and Oropuche guppies (*P. wingei, P. obscura*) indicating a common origin of the Y in this group. A recent molecular study in the swamp guppy (*Micropoecilia. picta*) reported a low SNP density on the Y, indicating Y-chromosome deterioration. We performed a series of cytological studies on *M. picta* to show that the Y-chromosome is quite small compared to the X and has accumulated a high content of heterochromatin. Furthermore, the Y-chromosome stands out in displaying CpG clusters around the centromeric region. These cytological findings evidently illustrate that the Y-chromosome in *M. picta* is indeed highly degenerated. Immunostaining for SYCP3 and MLH1 in pachytene meiocytes revealed that a substantial part of the Y remains associated with the X. A specific MLH1 hotspot site was persistently marked at the distal end of the associated XY structure. These results unveil a landmark of a recombining pseudoautosomal region on the otherwise strongly degenerated Y chromosome of *M. picta*. Hormone treatments of females revealed that, unexpectedly, no sexually antagonistic color gene is Y-linked in *M. picta*. All these differences to the *Poecilia* group of guppies indicate that the trajectories associated with the evolution of sex chromosomes are not in parallel.

## 1. Introduction

Unlike the highly conserved sex determination mechanism and sex chromosomes of birds and mammals, the poikilothermous vertebrates, reptiles, amphibians, and especially fishes have a remarkably wide range of sex determining mechanisms, including environmental sex determination [1,2,3]. The broad spectrum of genetic sex determining mechanisms is reflected in a high diversity of sex chromosomes offering important insights into the evolution of early sex chromosome differentiation and their turnover.

Historically, the common or Orinoco guppy, *P. reticulata,* has received great attention because many of the genes encoding the conspicuous coloration of males are Y-linked. Some of these Y-linked genes are strictly transmitted to male offspring only, while some color genes can cross-over to the X chromosome and vice versa. In addition, these Y-linked color genes are often polymorphic in the natural population and beneficial to males because they increase attractiveness to females [4]. The polymorphic Y-linked color genes are assumed to be sexually antagonistic, because they are beneficial to males but detrimental to females, not serving their attractiveness but making them more conspicuous to predators [5,6], in accordance with the hypothesis that sexually antagonistic genes for male coloration enrich on the Y chromosome [7]. However, the sexual conflicting situation does not manifest, because the color patterns are under strict hormonal control and are only expressed in males.

Early genetic studies in the common guppy established male heterogamety with extensive homology between the X and Y chromosomes and only a small differential sex specific region on the Y chromosome [8] that is expected to harbor the male sex determining locus. Although male guppies have long been known to be heterogametic, their sex chromosomes cannot be easily distinguished cytologically. Only heterochromatin staining could unveil an accumulation of heterochromatin at the terminal end of the long arm of the Y chromosome [9,10]. LG12 was identified as the putative sex chromosome [10] and subsequently verified through FISH analysis with BAC probes [11]. Recent molecular studies using linkage mapping and whole genome sequencing led to conflicting conclusions about the recombination rates over the length of LG12, the presence of strata, and the number and location of pseudoautosomal and male specific regions on the Y (MSY) [10,12,13,14,15,16,17,18,19,20,21]. Yet, despite intense research, the identification of X or Y-specific genes and of the sex determining gene of guppy remains elusive.

Besides the well-known common or Orinoco guppy, *P. reticulata*, the name “guppy” designates several small-sized Poeciliid species which share an extreme sexual dimorphism in pigmentation. They belong to two genera. The subgenus *Acanthophacelus* of the genus *Poecilia* includes the common or Orinoco guppy, *P. reticulata*, the Cumaná or Endler’s guppy, *P. wingei*, and the Oropuche guppy, *P. obscura* [22]. The closely related genus *Micropoecilia* consists of seven species, which include the swamp guppy, *M. picta*, and Pará guppy, *M. parae*, but also some species with a low degree of sexual dimorphism. Comprehensive FISH analysis with *P. reticulata* BACs from LG12 in several populations of *P. wingei* confirmed that the sex chromosomes of both guppies are homologous and carry a large recombining region [11] which has been corroborated through meiotic analysis [23,24]. A similar sex chromosome organization was demonstrated for *P. obscura* [11]. Thus, the ancestral LG 12 has been retained as sex chromosomes in all species of the subgenus *Acanthophacelus*. The sex chromosomes in the three guppies show a large euchromatic pseudoautosomal region (PAR). The terminal Y-specific heterochromatin can undergo enormous expansion in some populations of *P. wingei*, leading to an increase of Y chromosome size. However, cytological information on closely related *Micropoecilia* guppies, such as *M. parae* and *M. picta,* remained absent so far.

In a recent comparative study, homology between the sex chromosomal linkage groups of *P. reticulata, P. wingei,* and *M. picta* was shown. Interestingly, for LG12 of *M. picta*, there was a significantly lower SNP density than in *P. reticulata* and *P. wingei* and only half read coverage implying a hemizygous state of the X in males. This result led to the conclusion that the Y chromosome of *M. picta* is non-recombining and has undergone significant decay [14]. Such a situation raises interesting questions concerning the extent of Y chromosome deterioration and suppression of its recombination with X, which require cytological confirmation. A conflicting hypothesis about the evolution of the Y-chromosomes of guppies was put forward based on the absence of Y-X homologous genes divergence times estimates which postulates a sex chromosome turnover and the creation of a new Y from the X [25].

We performed a comprehensive cytogenetic analysis in *M. picta* to investigate the structure of the Y chromosome and the extent of its degeneration. The recombination status between the X and Y chromosomes was assessed through immunostaining with MLH1, a DNA mismatch repair protein involved in meiotic crossing over, and SYCP3, a component of the synaptonemal complex in zygotene and pachytene meiocytes. Additionally, indirect immunofluorescence using monoclonal anti-5-MeC antibodies to detect hypermethylated sites on the Y chromosome was performed. As the Y-chromosome of *M. picta* has been shown to be homologous to the sex chromosome of the common guppy [14,25], we wanted to elaborate if and how evolution of the degenerated swamp guppy Y affected the sexually antagonistic color genes. The presence of Y-linked pigmentation patterns was therefore checked by androgen treatment experiments of female guppies of the subgenus *Acanthophacelus* and of *M. picta* and a comparison of the induced pigmentation patterns to those of untreated and treated males.

## 2. Materials and Methods

### 2.1. Animals

All fish were reared under a standard conditions for Poeciliid fish husbandry [26] with a light/dark cycle of 14/10 h at 26 °C in the fish facility of the Biocentre at the University of Wuerzburg, Germany. All animals were kept and sampled in accordance with the applicable EU and national German legislation governing animal experimentation. In particular, all experimental protocols were approved through an authorization (568/300-1870/13) of the Veterinary Office of the District Government of Lower Franconia, Germany, in accordance with the German Animal Protection Law (TierSchG).

For details about the different fish strains used in this study, see Supplementary Table S1.

As a rare event, spontaneously XX males occur in *Acanthophacelus* species, which are easily recognized by their color pattern that deviates from normal males and matches the pigmentation of androgen-treated females. Such XX males of *P. wingei* were mated to normal females. Fish with a larger body size (n = 4) were infertile. Smaller XX males (n = 2) were fertile and produced all female offspring (40 and 154).

### 2.2. Mitotic Chromosome Preparation

Cytogenetic studies were conducted with the TR strain of *M. picta* and LP strain of *P. wingei*. At least 20 fishes from both sexes were analyzed.

Metaphase chromosomes from both males and females were obtained according to the standard procedure described previously [11]. Briefly, pooled soft tissues (cephalic kidney, gill, spleen, and intestine) were minced in a hypotonic solution (0.46% KCl) after exposing fishes to a 0.02% solution of colchicine for 8–10 h. It was necessary to pool tissues from 5–6 individual fishes to obtain a reasonable number of analyzable metaphases. In our experience, the preparation of chromosomes from a single individual of small size poecilid fishes does not yield good numbers of metaphases as most metaphase chromosomes appear overlapping. The cell suspension was incubated in a hypotonic solution for 30 min at room temperature and fixed with a mixture of methanol and acetic acid (3:1). After overnight fixation, slides were prepared and stained with 5% Giemsa solution. Additionally, several slides were stained with DAPI (4′-6-diamidino-2-phenylindole). To stain the constitutive heterochromatin, slides were subjected to C-banding using the standard BSG (barium hydroxide/saline/Giemsa) technique [27].

### 2.3. Meiotic Chromosome Preparation

Analysis of meiotic chromosomes was performed using the standard direct preparation. Synaptonemal complexes (SCs) analysis was performed in zygotene-pachytene meiocytes. For the direct preparation, seminiferous tubules were dissected from the testes of mature males. Seminiferous tubules were kept in a hypotonic solution for 30 min at room temperature and transferred to fixative solution. To obtain meiotic cells, pieces of seminiferous tubules were placed on a slide and cells were mechanically released and spread with a Pasteur pipette at 40 °C on a hot plate in a small drop of 50% acetic acid. After the complete evaporation of acetic acid, slides were stained with Giemsa solution and meiotic stages were analyzed under a light microscope. Constitutive heterochromatin on meiotic chromosomes was visualized through C-banding.

To visualize SCs, small pieces of seminiferous tubules were first minced in a small drop of hypotonic solution (0.46% KCl) and the released cells were left in the hypotonic solution for at least 20 min. One drop of cell suspension was placed on a clean slide and was mixed with three drops of 2% paraformaldehyde by gentle shaking of slides. After 10 min of fixation, fresh drops of paraformaldehyde solution were added to the slides, followed by a brief washing of slides in 0.4% Photoflo. Slides were checked under a phase-contrast microscope to monitor the quality of SC spreads. Staining of SC spreads was achieved by the ammoniacal silver (Ag-AS) method of [28].

### 2.4. Immunostaining of Synaptonemal Complex and MLH1

Synaptonemal complexes (SC) and MLH1 sites during pachytene were simultaneously visualized using immunofluorescent staining of slides containing spreads of meiocytes with slight modification of the protocol described [24]. Synaptonemal complexes were detected by in-house generated Rabbit polyclonal antibodies against the lateral component of SC SYCP3 (1:250; [29]) with goat-anti Rabbit-Alexa 594 secondary antibodies (1:200, Thermo-Fisher Scientific Inc., Waltham, MA, USA) and a mouse monoclonal antibody to mismatch repair protein MLH1 (1:30; Abcam; ab14206, Cambridge, UK) with goat anti-mouse FITC-conjugated secondary antibodies (1:30; Jackson ImmunoReseach, Cambridgeshire, UK) was used to recognize the MLH1 sites. All antibodies were diluted in PBT (3% bovine serum albumin and 0.05% Tween 20 in 1xPBS). A solution of 10% PBT was used for blocking unspecific antibody binding. Primary antibody incubation was carried out overnight in a humid chamber at 37 °C, followed by secondary antibody incubation for 1 h at 37 °C. After a brief wash in PBS, slides were mounted in Vectashield with DAPI (Vector Laboratories) to stain DNA and reduce fluorescence fading.

Signals for both antibodies were separately photographed using a Zeiss Axio Imager (Carl Zeiss, Oberkochen, Germany) M1 microscope equipped with CCD camera. Individual images showing specific immunofluorescent signals were merged using the ASI (Applied Spectral Imaging Ltd., Yokneam, Israel) software (FISHView 6.0).

### 2.5. Immunolocalization of Anti-5-Methylcytosine

An indirect immunofluorescence approach using a monoclonal antibody against 5-MeC, as described in detail in [30], was employed to detect the hypermethylated regions on fixed metaphase chromosomes.

### 2.6. Hormone Treatments

Testosterone treatments were performed by adding 100 µL of 17-methyl-testosterone stock solution (10 mg/mL ethanol) to 5-L tanks (without water filtering) each Monday, Wednesday and Friday around 8 am. From each strain, 10 fish were treated. During weekends, gravel filters were reintroduced and water changes were performed before restarting treatments the following week. Fish were treated until no further changes in coloration occurred for at least two weeks. On average, the treatments lasted for 12 weeks.

## 3. Results

### 3.1. Cytological Identification of the M. picta Y Chromosome

**Figure 1:**
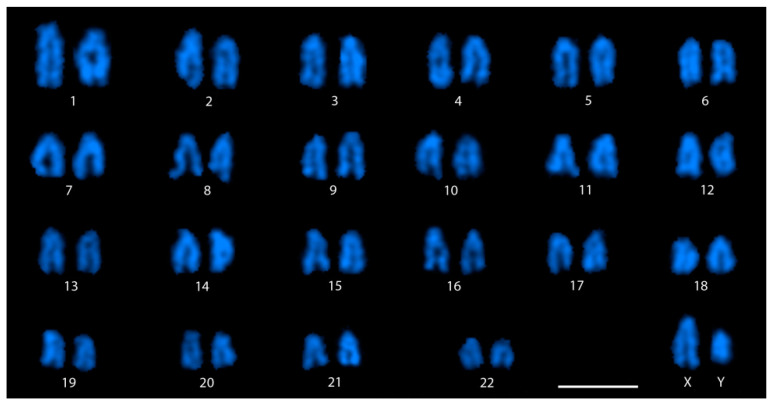
DAPI stained karyotype of male *M. picta*. Note the size difference between the X and Y. The chromosomes are arranged roughly based on their length. Bar: 3 µm.

In species with highly differentiated sex chromosomes, the heterogametic sex specific chromosome (Y or W) exists as an odd heteromorphic chromosome compared to autosome pairs, allowing its easy identification in conventional karyotype analysis. Simple Giemsa staining and counting of chromosomes in well spread metaphases from both sexes of *M. picta* revealed a diploid chromosome number of 46, and all chromosomes appear to be acrocentric, just as in other related guppy species. Karyotyping at least five complete DAPI stained metaphases failed to find equal sized homologues for large and small acrocentric chromosomes only in males (Figure 1). Since the large acrocentric chromosome can be arranged easily with its homolog in females (Supplementary Figure S1), the large and small acrocentric chromosomes without their homologues marked in the male karyotypes can thus be assigned as X and Y, respectively.

The DAPI stained karyotype (Figure 1) presenting the staining pattern of each pair of homologous chromosomes shows that the Y chromosome in *M. picta* is distinctly much smaller than the X chromosome. It should be specified that the X chromosome size appears to be of similar size with many of the large acrocentric chromosomes (chromosome pairs 2 to 5). No remarkable staining pattern that could point to the accumulation of heterochromatin on specific chromosomes was observed through DAPI staining.

**Figure 2:**
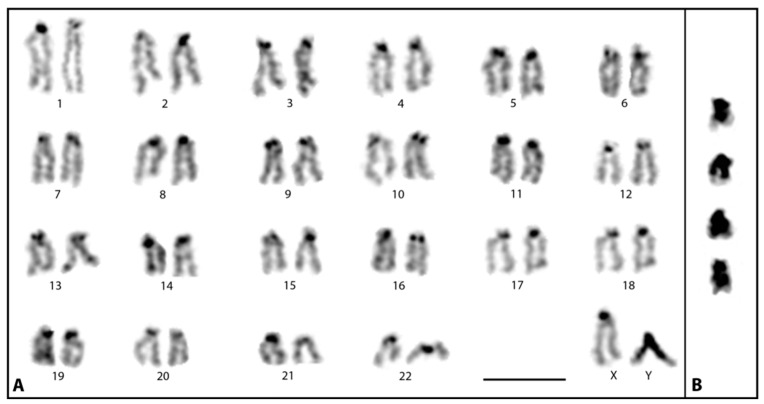
Heterochromatin staining of *M. picta* metaphase chromosomes. (**A**) C-banded karyotype of male displaying the heterochromatic region on the Y chromosome. (**B**) C-banded Y chromosome cut-outs from different metaphases. Note a short non-heterochromatic region at the very distal region of the long arm of the Y. Bar: 3 µm.

Since different amounts of heterochromatin accumulation on Y chromosomes was previously reported in closely related *Poecilia* guppy species [11], heterochromatin staining was performed on metaphases of *M. picta* by C-banding. Essentially, heterochromatin staining on autosomes and the X chromosome was restricted to centromere regions. In addition, a few chromosomes were found to have a small heterochromatic region close to the long arm telomeres. However, the staining pattern on the Y chromosome stood out because the heterochromatin specific staining was visible roughly all along its length except the very distal end of the long arm (Figure 2A,B). The staining pattern of this region is comparable to the euchromatic regions on other chromosomes, suggesting that the small terminal region of the Y chromosome may not be heterochromatic. In extremely condensed metaphases, the negative heterochromatic staining at the terminal region of the Y may not be very conspicuous. This characteristic negative heterochromatin staining at the short distal segment of the Y was consistently marked in all metaphases analyzed (>50 metaphases). Since our analysis was performed on a pool of several individuals in each preparation, a polymorphism in the staining pattern involving this short terminal region of the Y would have been detected and thus can be ruled out.

### 3.2. Meiotic Association and Recombination between the X and Y

**Figure 3:**
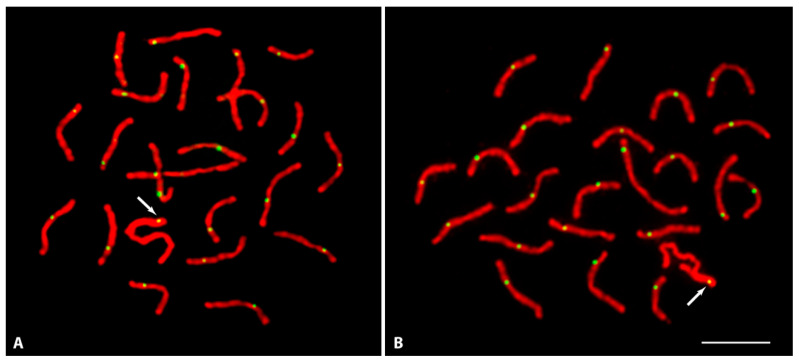
Meiotic spread of testis displaying complete set of autosomal synaptonemal complexes and the sex pair after immunolocalization of SYCP3 (red) and MLH1 (green) at pachytene stage. The green MLH1 specific spot on each synaptonemal complex points to the recombination sites. The arrows show the single MLH1 site located at the distal end of the XY complete (**A**) or partial (**B**) synaptic region. Bar: 3 µm.

Investigation on synapsis and recombination between X and Y during meiosis sheds special light on recombination suppression. Based on the degree of homology, synapsis between the sex chromosomes can be recognized under the light microscope through silver-staining of zygotene-pachytene meiocytes or at a higher resolution by the immunostaining of synaptonemal complex proteins. Immunostaining with SYCP3 antibodies was unambiguously able to detect all 22 SCs, each corresponding to an individual autosomal bivalent plus the XY structure (Figure 3). The XY pair can be easily differentiated from the autosomal SCs because a large part of the X axial element appears extended, indicating an unpaired structure. In pachytene stage, both X and Y were found to be fully associated or partially associated (Figure 3A,B). In the latter, the partial association may suggest a synapsis between the X and Y. In over 200 pachytene meiocytes analyzed, in no case were the X and Y chromosomes observed as separate entities. A similar association between the X and Y was also noticed in silver-stained surface spread meiocytes (Supplementary Figure S2).

Recombination sites on each bivalent were detected through the immunolocalization of MLH1, which marks the sites where homologous chromosomes have undergone (or are about to undergo) reciprocal recombination (i.e., the sites of crossovers or chiasmata). MLH1 foci were observed in each SYCP3 stained autosomal SC and the XY structure (Figure 3). Each autosomal SC displayed a single MLH1 site. On average, 18–21 MLH1 sites were observed in all analyzed cells. Interestingly, a single MLH1 signal was consistently marked close to the very distal part of associated region of the XY. In both fully associated or partially associated X and Y the single MLH1 site was always confined to the same region. MLH1 staining on the XY structures was analyzed only in those cells displaying MLH1 sites on all autosomal SCs. Cells without MLH1 signals on a single or several autosomal SCs were not considered, as they might present regional problems of accessibility for the antibodies. Of over 100 pachytene stages, which were analyzed in the study for MLH1 staining, 18 pachytene meiocytes showed MLH1 signals on every autosomal pair and all these 18 cells also displayed a positive MLH1 signal on the XY pair.

Since the distal region of the long arm of the Y is not heterochromatic (Figure 2), we assume that the observed MLH1 site on the XY pair may very well coincide with the distal non-heterochromatic region of the Y.

**Figure 4:**
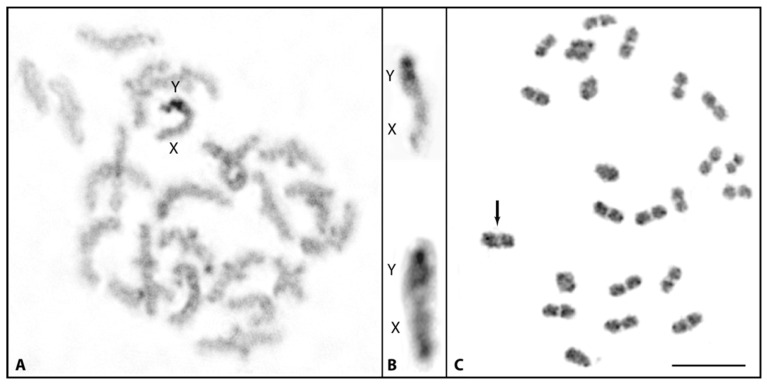
C-banded meiotic guppy chromosomes. Diakinesis-metaphase I (**A**) and XY cut-outs (**B**) of *M. picta* showing end-to-end association between the X and Y during meiosis. (**C**) Metaphase I of *Poecilia wingei* (LP strain). Note the association between the X and Y (arrow) appears to be similar with autosomal bivalents. Bar: 3 µm.

Meiotic analysis was further extended to C-banded diakinesis–metaphase I stages to verify the association between X and Y which was evident by the presence of MLH1 foci on the sex chromosomes for their association being mediated by a chiasma. Due to the heterochromatic nature of the Y, it was easy to identify in the XY bivalent (Figure 4A). In all diakinesis–metaphase I analyzed, the X and Y chromosomes always remained terminally associated (end-to end), indicating no typical chiasmatic association as it is seen among autosomal bivalents (Figure 4A). Since the centromeres are positioned in opposite orientation in the sex chromosome bivalents (see cut-outs, Figure 4B), the end-to-end association between the X and Y chromosomes is confined to their long arms distal end. For comparison, meiotic analysis was performed in male *P. wingei*. In this species, the Y chromosome is extremely large with a sizable amount of heterochromatin and association of the sex chromosomes is terminal. This is in agreement with findings showing recombination to occur at the distal end between X and Y in *Poecilia* guppies [20]. Compared to *M. picta*, the association during metaphase I between X and Y in *P. wingei* is rather different. Though it appears to be end-to-end (Figure 4C), the association intersection looks almost diffuse (less stained), like in autosome bivalents, indicating terminal chiasmata.

### 3.3. Y-Specific Hypermethylated Region

**Figure 5:**
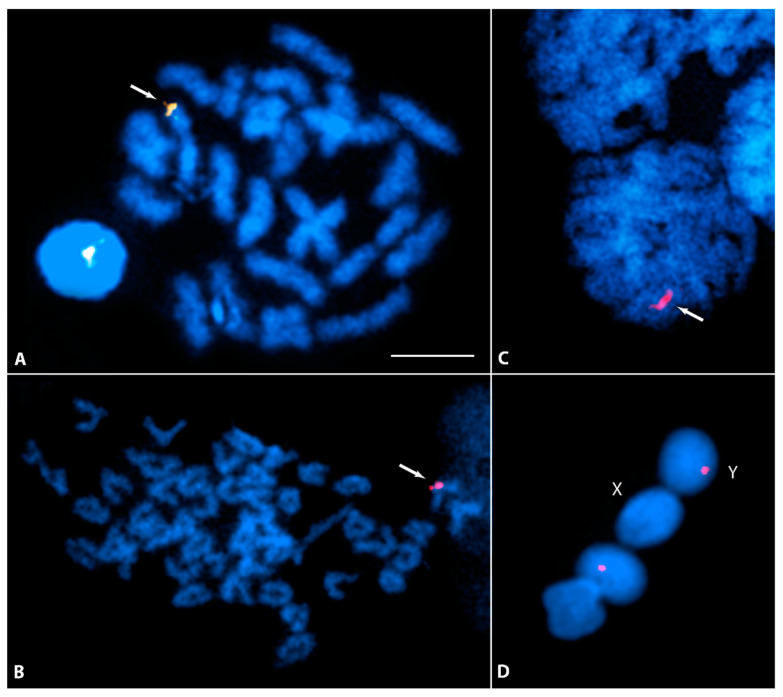
Indirect immunofluorescence showing 5-methylcytosine rich regions in metaphase I (**A**) and somatic male metaphase (**B**) of *M. picta*. The strong signal of hypermethylated sites marked by red fluorescence is specifically located around the centromeric region of the Y chromosome (arrows). Chromosomes are counterstained with DAPI. The strong fluorescence of the 5-MeC-region can be visualized (arrows) in pachytene (**C**), and Y bearing spermatid (**D**). Bar: 3 µm.

DNA-methylation, a stable epigenetic signature, is known to be essential for heterochromatin formation and maintenance. Correspondingly, the immunostaining of anti-5-MeC antibodies in many species has shown hypermethylated regions restricted to constitutive heterochromatin. Anti-5-MeC antibodies staining initially carried out on metaphase I chromosomes of *M. picta* detected a bright staining invariably at one end of a bivalent (Figure 5A), indicating that the region highlighted by anti-MeC antibodies might be located on the Y chromosome. To confirm the Y-chromosome staining, male mitotic metaphases were stained with anti-5-MeC antibodies which showed strong fluorescent signal on a single chromosome. Since no chromosome was similarly labelled with the antibody in female metaphases, the chromosome strongly labelled with anti-5-MeC antibodies in male metaphases was evidently the Y chromosome (Figure 5B). The strong anti-5-MeC staining mostly covers the centromeric region. However, based on the intensity of the signal, anti-5-MeC staining appears to extend to the proximal region of the centromere toward the telomere. Strikingly, this specific staining on the Y chromosome of *M. picta* can be visualized in pachytene stage chromosomes as well as in round nuclei (likely early spermatids, Figure 5C,D).

Remarkably, anti-5-MeC antibodies labelling was absent from the euchromatic regions of the autosomes. Moreover, 5-MeC staining visible on the centromeres or telomeres of autosomes was significantly less intense. No such specific 5-MeC staining was observed for the Y chromosome of *P. wingei* (Supplementary Figure S3).

### 3.4. Y Chromosome-Linked Pigmentation Patterns

**Figure 6:**
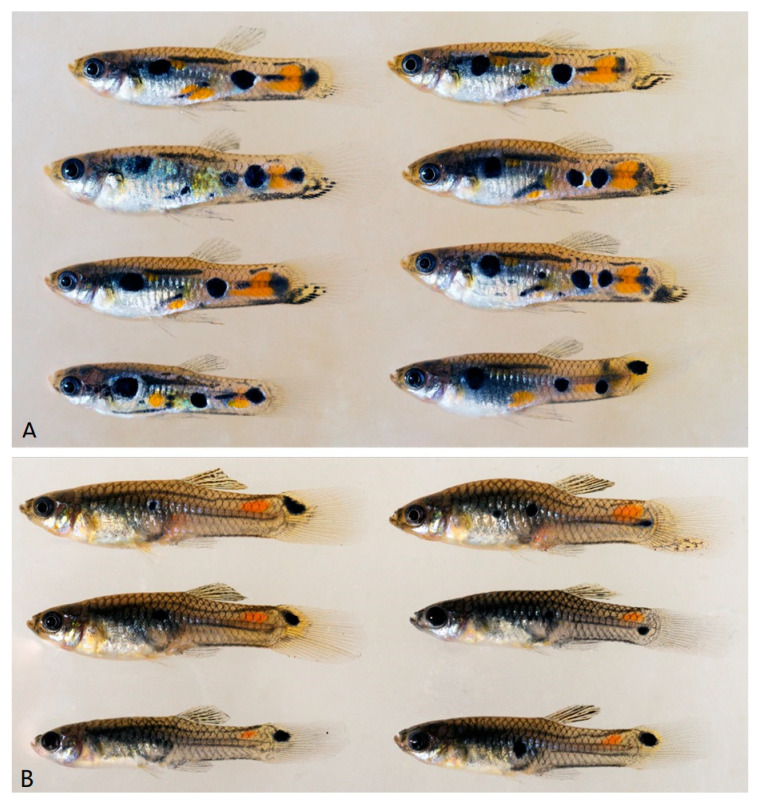
Non-treated Males (**A**) and females (**B**) treated with 17-methyltestosterone of *P. obscura* (RS strain). Hormone treated females are showing only a part of the male pigmentation patterns. For the phenotype of untreated females see Supplementary Figure S17.

**Figure 7:**
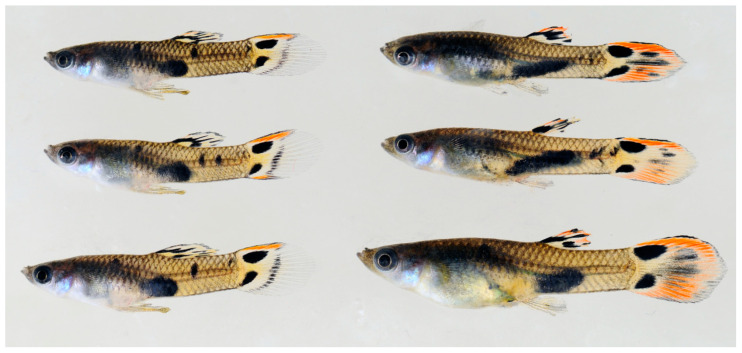
Untreated males (**left**) and 17-methyltestosterone treated females (**right**) of *M. picta* (FG strain). Hormone treated females show the complete male pigmentation pattern. For the phenotype of untreated females see Supplementary Figure S17.

Androgen treatment of XX females and comparing their pigmentation phenotypes to XY males enables to determine which components of the genetic information for the pigmentation pattern are encoded on the Y chromosomes. Pigmentation that develops in the treated females is not due to Y-linked genes, but due to X-linked or autosomal color genes. We treated XX females of several strains of *P. reticulata* (n = 6), *P. wingei* (n = 4), *P. obscura* (n = 2), and *M. picta* (n = 2), recorded their pigment patterns after long term treatment, and compared them to males of the same strain (Figure 6 and Figure 7 and Supplementary Figures S4–S16).

All testosterone treated females of the *Poecilia* guppies developed some of the pigment patterns typical for males, although to a different degree, depending on the strain. In no case did all pattern components expressed in males develop in the XX fish, allowing to determine which part of the male coloration is encoded in the MSY. This result was confirmed by the phenotype of seven spontaneously occurring XX males of *P. wingei* (Supplementary Figure S15).

Conversely, in both strains of *M. picta*, the pigmentation pattern of the testosterone treated XX females was identical to those of XY males (Figure 7 and Supplementary Figure S16). This shows that the large MSY of *M. picta* does not encode information for the typical male coloration.

## 4. Discussion

**Figure 8:**
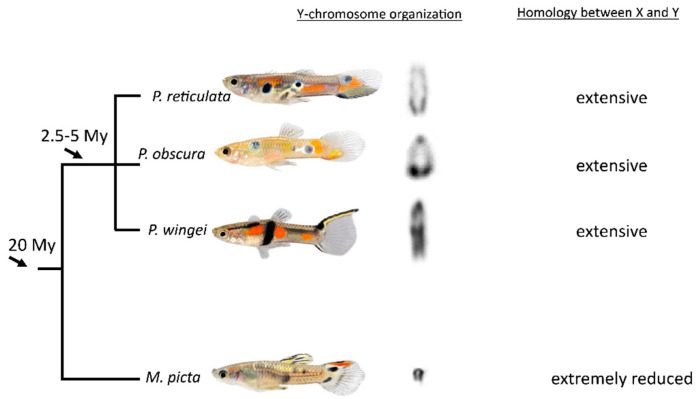
Dynamic evolutionary transition of Y-chromosome structure among related guppies. The cut-outs of the Y-chromosomes are from the same magnification. The phylogenetic relationship was arranged according to [11,18].

Traditionally, homomorphic (or almost homomorphic) sex chromosomes are considered to represent the early stage of evolution of highly differentiated heterogametic sex chromosomes of old lineages, as they undergo genetic recombination and the Y chromosome is not radically degenerated. These primeval evolutionary features are conspicuously preserved in the sex chromosomes of the *Acanthophacelus* guppies [11,13,17,18,31]. In a comprehensive genome analysis, intriguingly, extreme degeneration of the Y chromosome was recognized in the swamp guppy [14]. A recent study found also in the closely related Pará guppy, *M. parae*, a strong molecular degeneration of the Y chromosome [32]. Along this line of evidence, the present cytogenetic study confirms the substantial degree of decay of the heterogametic sex chromosome of *M. picta* by demonstrating a predominantly heterochromatic Y. The molecular degeneration of the Y chromosome has also led to an extreme size reduction to much less than the size of the X. Comparing the cytogenetic features of mitotic and meiotic sex chromosomes of the four guppies studied so far clearly indicates that the Y chromosome in the swamp guppy *M. picta* is very different from *P. reticulata*, *P. wingei,* and *P. obscura* (Figure 8). It is small in size and strongly heterochromatic, while the Ys of the *Acanthophacelus* guppies are large and not smaller than the X [11]. The heterochromatin in these species is confined to a telomere-near block of differing size between species and populations.

Besides its small size and extensive heterochromatization, another feature of the *M. picta* Y is intriguing where it is also strikingly different from the Ys of *P*. *reticulata*, *P. wingei,* and *P. obscura*. For example, hypermethylated sites on the Y centromere of *M. picta* can be demonstrated with anti 5-MeC staining. Such a hypermethylated region is apparently absent from the Y chromosome of *P. wingei*. Anti 5-MeC staining highlighting the heterochromatin of sex chromosomes has been demonstrated in several fish species [30] and also on the human Y heterochromatin [33]. It should be noted that, unlike the preferential location of hypermethylated sites on sex chromosome heterochromatin in other species, in *M. picta*, the heterochromatin all along the Y remains largely undetected by anti 5-MeC staining. DNA methylation of repetitive elements is important to adopt a highly condensed chromatin structure [34] and to prevent retrotransposon activity [35]. Similarly, the methylation of the gene-regulatory region, in particular promoters, is associated with a repressive state [36]. The methylation of neighboring CpGs is highly interdependent and epigenetic silencing is usually mediated by methylation changes at the regional level. In light of this, it seems plausible to assume that hypermethylation of the (peri)centromeric region of the Y is involved in maintaining a highly condensed chromosome structure at the centromere and to distinguish X and Y chromosomes.

A quite interesting observation in this study was that the X and Y chromosomes of *M. picta*, though previously reported as non-recombining [14], in fact do show sites of late recombination. This is evidenced by the presence of MLH1 signals at the distal regions of the paired XY chromosomes in pachytene stage. MLH1 is a key component of the meiotic recombination machinery, required to transform late recombination events into functional cross-over sites [37]. In mammals, meiotic chromosomes require at least one to two MLH1 sites to ensure chiasma formation, which in turn is a prerequisite for the correct segregation of meiotic chromosomes. This also includes the XY chromosome pair that necessarily shows MLH1 foci in the pseudoautosomal region (PAR) of the synapsed pachytene chromosome regions, where crossing over is known to occur [38]. Therefore, the observed MLH1 sites in *M. picta* at the distal end of the XY pair undoubtedly mark the presence of a synaptic region between the XY pair that could resemble a classical PAR as seen in mammals and even fishes, e.g., the guppies of the of the subgenus *Acanthophacelus* [24,37,38]. Based on the extensive analysis of coverage and distribution of SNPs on guppy sex chromosomes [14], it was suggested that the degenerated Y chromosome in *M. picta* is completely non-recombining with the X. Our results, however, show that there is a pseudoautosomal region at the distal end of the Y-chromosome long arm. According to C-banding (Figure 2), the PAR is at least several megabases in size and thus larger than predicted from the SNP analysis [14]. Clearly, the presence of MLH1 signals in this region indicates recombination.

The fact that the Y can still align over almost its entire length is an indication that the differentiation noted in the SNP analyses does not create a high enough sequence divergence of what is designated “sex-chromosome specific region” to interfere with synapsis.

A comparison of SNP densities and male to female read coverage between the X and Y chromosomes revealed that the very terminal region showed an increase of density and coverage close to the normal range, which allowed Darolti et al. [14] to anticipate the existence of a PAR. The association between X and Y detected in our study through SYCP3 immunostaining and localization of MLH1 in the associated region of XY structure directly visualizes the existence of a PAR in *M. picta* at the distal end that should be important for the recombination and proper disjunction of the sex chromosomes. In the C-banded mitotic metaphases (Figure 2), the very distal end of the Y chromosome appears to be less stained, suggesting that this distal non-heterochromatic region on the Y most likely corresponds to the PAR visible in the XY structure in the pachytene stage. In contrast to the more cryptic situation in *M. picta*, in *Acanthopacelus* guppies, both X and Y, which during meiosis undergo recombination at their distal end [24], form a chiasma-like structure between each other, as can be clearly seen for example at metaphase I stage in *P. wingei* (Figure 4C).

The color pattern and fin shapes of wild *P. reticulata* males are encoded by a suite of dominant loci that encode certain extensions of the dorsal and tail fin and spot or stripes on the body and fins. Genetic studies revealed that at least one, but generally several of these loci contribute to the color and fin dimorphism [8,39]. Some loci are Y-specific, while others can undergo sex chromosomal crossing over and can be located either on the X or the Y [40]. Only a small number of color and fin loci are autosomal. Our treatment experiments confirmed the presence of pigmentation loci outside the Y-specific region by showing that XX females of *P. reticulata* can express after testosterone induction a subset of the coloration and the fin morphologies of males from the same strain. The same result was obtained for the two other species of the subgenus. Moreover, spontaneous sex-reversed XX males of *P. wingei* exhibited part of the male pigmentation pattern. We observed that the fraction of the full male coloration encoded in the female genome differed between strains. This is in accordance with a testosterone treatment study of wild *P. reticulata*, which showed different degrees of MSY encoded color patterns between fish from high and low predation sites [41]. However, for all three *Acanthophacelus* guppies, it was confirmed that a considerable part of the male coloration is encoded from loci of the MSY.

Conversely, the testosterone treated females of *M. picta* developed the full male pigmentation pattern, showing that the Y-chromosomes of this species do not harbor pigmentation genes. Sexually antagonistic genes are regarded as strong drivers of sex chromosome evolution and the suppression of recombination [7]. It was therefore unexpected that, in *M. picta*, the highly degenerated Y chromosome is not the location of the only so far known sexually antagonistic trait in this species. Unlike in the *Acanthophacelus* guppies, there is little coloration polymorphism in *M. picta* males, and the sex-limited expression of the male pigmentation pattern has reduced any sexual conflict, which may provide a possible explanation for the non-linkage. One may speculate that the absence of color-related loci on the Y-chromosome of *M. picta* is a consequence of its genetic degeneration. Given the heavily debated and unresolved evolutionary history of the sex chromosomes in guppies [14,21,25], a similar study to the one conducted here on *M. picta* in the sister species *M. parae* is inevitable. Studies with natural populations are necessary to perform ecological and evolutionary analyses concerning population sizes and the fixation of structural chromosome alterations.

It has been shown that the X-chromosomes in *P. reticulata*, *P. wingei*, and *M. picta* share the same ancestral X [14]. Since the divergence between *M. picta* and the related two guppies is not more than 10 Mys, the Y chromosomes have obviously undergone a different fate in the *Micropoecilia* and *Acanthophacelus* lineages. This has been explained by a different speed of decay and degree of recombination suppression [14]. Alternatively, a “rejuvenating” homologous turnover in the *Acanthophacelus* lineage is also possible [25]. To date, the data availability remains too limited to make definitive conclusions about the origin of the Y-chromosomes in both lineages [14,21,25]. With respect to sex chromosome evolution in guppies and reconstruction of the ancestral state, the lack of pigmentation genes from the *M. picta* Y should be seen in the context of the prediction that the conditions for a sex chromosome turnover are less restrictive when sexually antagonistic genes have not yet accumulated on the ancestral Y [42].

## Data Availability

All data generated or analyzed during this study are included in this article and its supplementary material files. Further enquiries can be directed to the corresponding author.

## Supplementary Materials

The following supporting information can be downloaded at: https://www.mdpi.com/article/10.3390/cells11071118/s1, Figure S1: C-banded karyotype of female *M. picta*, Figure S2: Silver-stained meiotic prophase displaying autosomal SCs and the XY synapsis, Figure S3: Indirect immunofluorescence showing 5-methylcytosine rich regions, Figure S4: Males (upper) and testosterone treated females (lower) of *P. reticulata*, strain RR, Figure S5: Males (upper) and testosterone treated females (lower) of *P. reticulata*, strain “pauper”, Figure S6: Males (upper) and testosterone treated females (lower) of *P. reticulata*, strain IstW, Figure S7: Males (upper) and testosterone treated females (lower) of *P. reticulata*, strain ma-ze, Figure S8: Males (upper) and testosterone treated females (lower) of *P. reticulata*, strain HR, Figure S9: Males (upper) and testosterone treated females (lower) of *P. reticulata*, strain HR, Figure S10: Males (upper) and testosterone treated females (lower) of *P. obscura*, strain OR, Figure S11: Males (upper) and testosterone treated females (lower) of *P. wingei*, strain Ca, Figure S12: Males (upper) and testosterone treated females (lower) of *P. wingei*, strain LP, laboratory line 3521, Figure S13: Males (upper) and testosterone treated females (lower) of *P. wingei*, strain LP, laboratory line 3521b, Figure S14: Males (upper) and testosterone treated females (lower) of *P. wingei*, strain LP, laboratory line 5898, Figure S15: XX males of *P. wingei.* (a) strain LP (b–d) strain Ca, Figure S16: Males (upper) and testosterone treated females (lower) of *M. picta*, strain TR, Figure S17: Untreated females of *P. wingei* (upper), *P. reticulata* (middle), *M. picta* (lower), Table S1: Origin of fish strains.

## Abbreviations

KCl	potassium chloride
5-MeC	5-methylcytosine
SC	synaptonemal complex
Ag-AS	silver-ammoniac silver
SNP	single-nucleotide polymorphism
CpG	5′—cytosine—phosphate—guanine—3′
LG12	linkage group 12
MSY-	male-specific region on Y
C-band-	centromeric band
