# Supplementary material for: Evolution of the Degenerated Y-Chromosome of the Swamp Guppy, Micropoecilia picta
Source: Cells. 2022 Mar 25;11(7):1118. doi: 10.3390/cells11071118 (PMC8997885; doi:10.3390/cells11071118)
Supplement: Supplementary file 1 [file cells-11-01118-s001.zip › cells-1594332 supplymentary after revision final/cells- 1594332 supplymentary table after revision.pdf]

Supplementary Table S1: Origin of fish strains

**Strain name**	**Strain ID (WLC#)**	**Geographical Origin**	**Remarks**
P. obscura OR	3492	Oropuche River (N 10° 43.052'; W 61° 8.871')	Cumaca Caves, Trinidad. Locus typicus of *P. obscura*, collected by S. Schories and M.Schartl
P. obscura RS	3507	Río Seco River	below Río Seco waterfall, at Salybia River junction, Trinidad
P. wingei Ca	3614	Campoma, between Laguna Buena Vista and Laguna Campoma	3 km north of Cariaco, Venezuela. Locus typicus of *P. wingei*; collected by F.N. Poeser
P. wingei LP	3521<br>3521b<br>5898	Laguna de los Patos (N 10° 25.043'; W 64° 12.065')	West of Cumaná, Venezuela
P. reticulata HR	3491	Hollis Reservoir (N 10° 39.760′ W 61° 11.734′)	Entrance of Hollis Reservoir at Mt. Carmel Ave, Trinidad
P. reticulata GR	5856	Lower Guanapo River	Twin Bridge, North West Trinidad, Tübingen genome strain, collected by D. Reznick.
P. reticulata RR	5000	Rojas River (N 10° 04.332' W 61° 30.192') Trinidad	Small ditch close to mouth into Quinam Bay, east of recreational park shore
P. reticulata ma-ze	6187	unkown	Laboratory strain "maculatus-zebrinus", established by O. Winge
P. reticulata pauper	1307	unkown	Laboratory strain "Pauper", established by O. Winge
P. reticulata IstW	1312	unknown	Laboratory strain "Istambul wild", established by K.Kosswig
M. picta FG	4333	Lac du bois diable, Kourou; French Guyana	collected 2009 by fish hobbyists
M. picta TR	5001	San Fernandez,Trinidad (N 10° 14.706' W 61° 30.292')	Brackish roadside ditch of the coastal highway
