# Supplementary material for: Evolution of the Degenerated Y-Chromosome of the Swamp Guppy, Micropoecilia picta
Source: Cells. 2022 Mar 25;11(7):1118. doi: 10.3390/cells11071118 (PMC8997885; doi:10.3390/cells11071118)
Supplement: Supplementary file 1 [file cells-11-01118-s001.zip › cells-1594332 supplymentary after revision final/Nanda et al.-Supplementary figures1-17-revised.pdf]

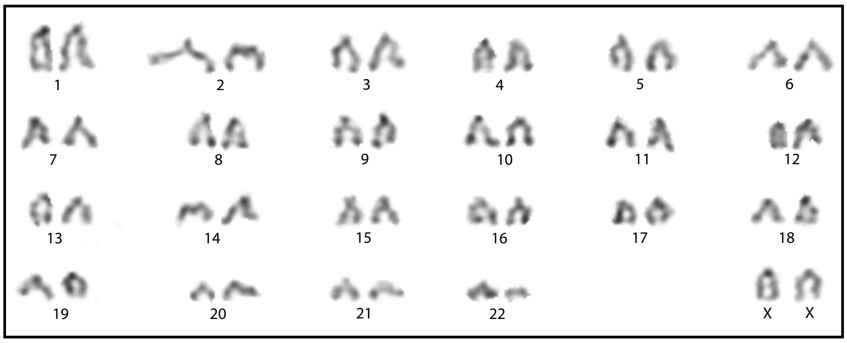

Supplementary Figure S1: C-banded karyotype of female *M. picta*

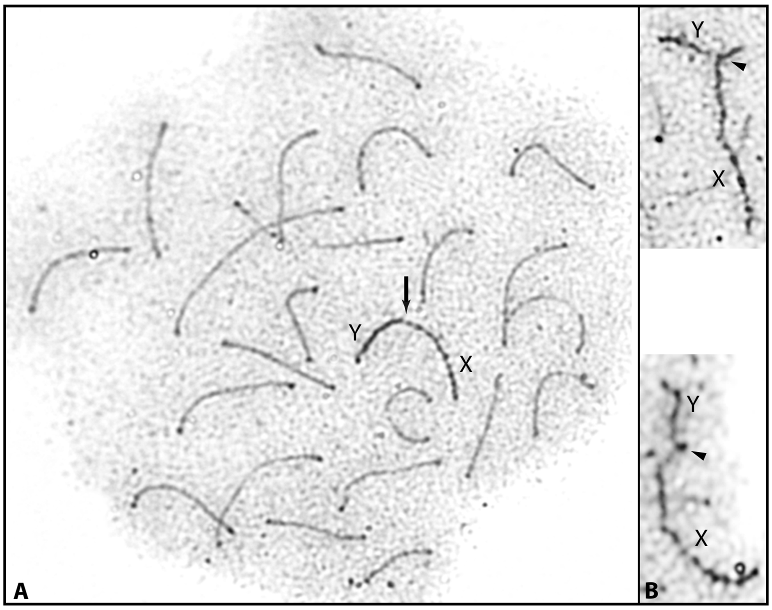

Supplementary Figure S2. Silver-stained meiotic prophase displaying autosomal SCs and the XY synapsis (**A**). Arrow indicates the full synapsis of Y with the X. (**B**) XY cutouts from pachytene showing partial paired associations (arrows).

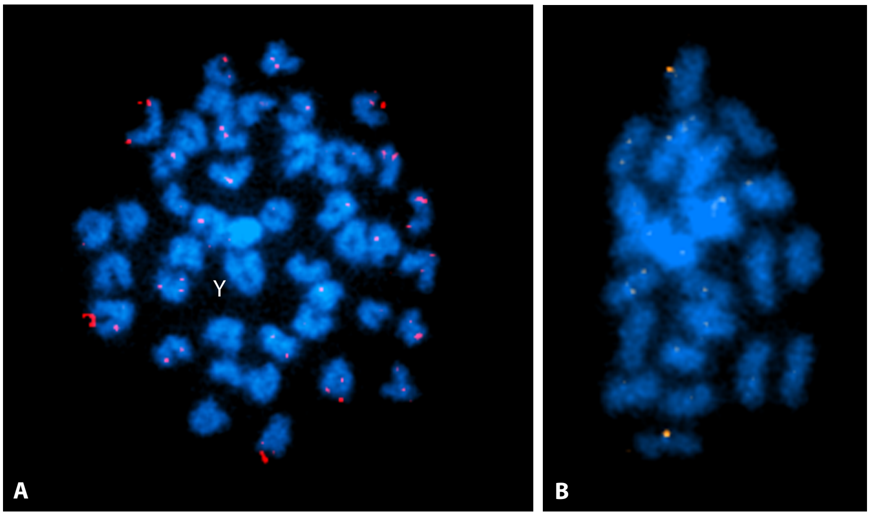

Supplementary Figure S3: Indirect immunofluorescence showing 5-methylcytosine rich regions in somatic metaphase (**A**) and metaphase I (**B**) of *P. wingei* (VE11). Note the absence of specific hypermethylated sites on the Y.

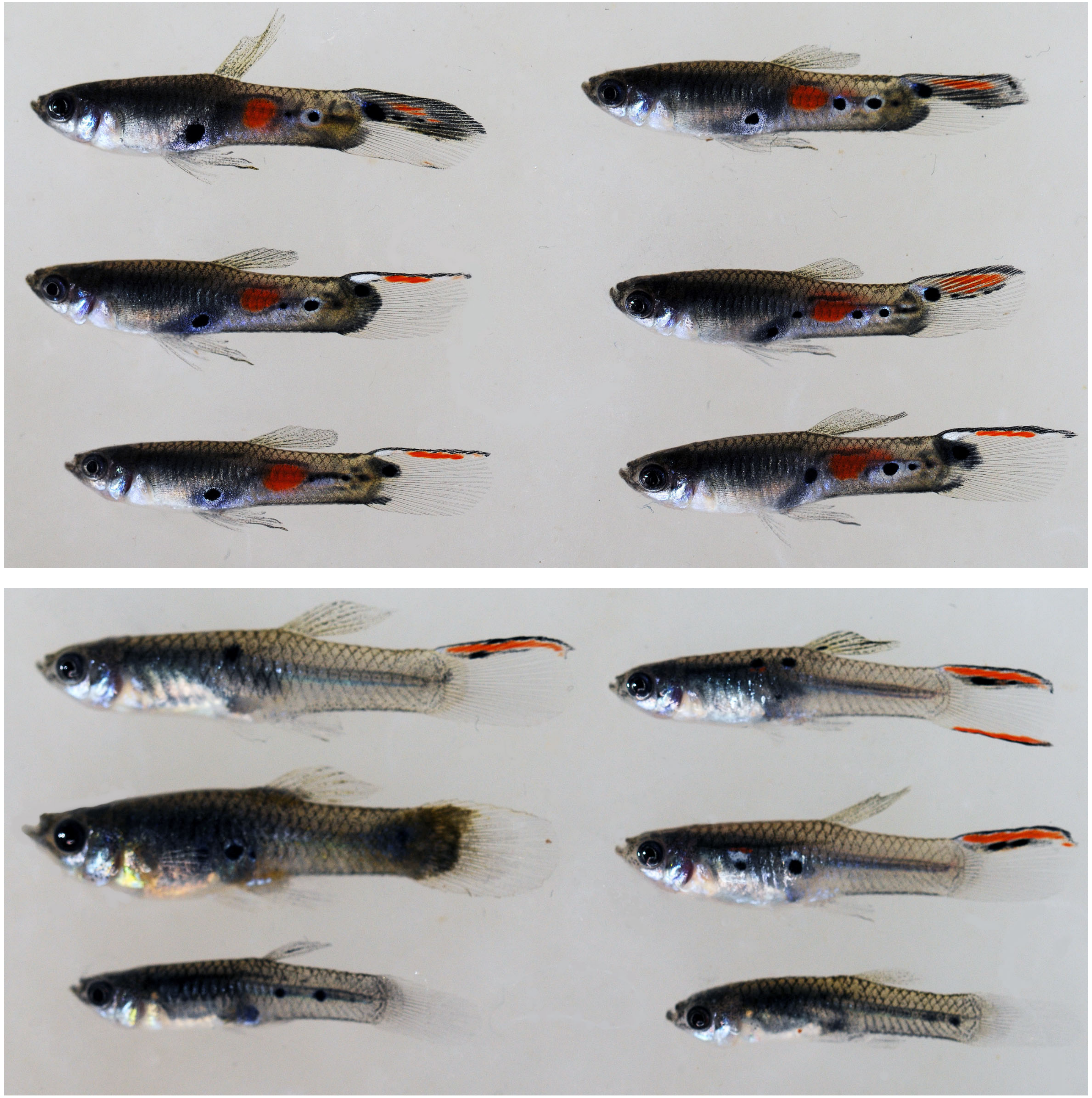

Supplementary Figure S4: Males (upper) and testosterone treated females (lower) of *P. reticulata*, strain RR.

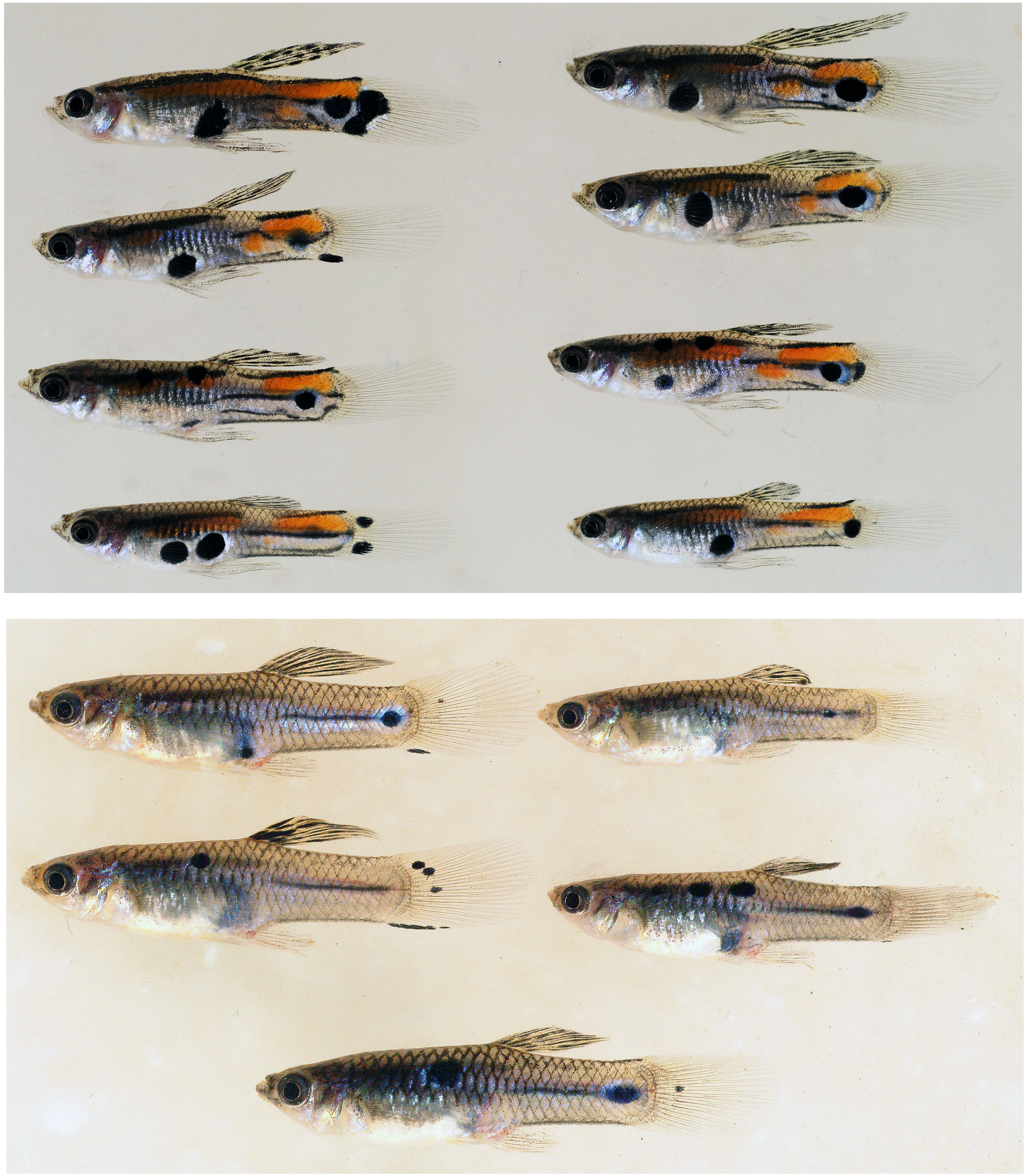

Supplementary Figure S5: Males (upper) and testosterone treated females (lower) of *P. reticulata*, strain „pauper“

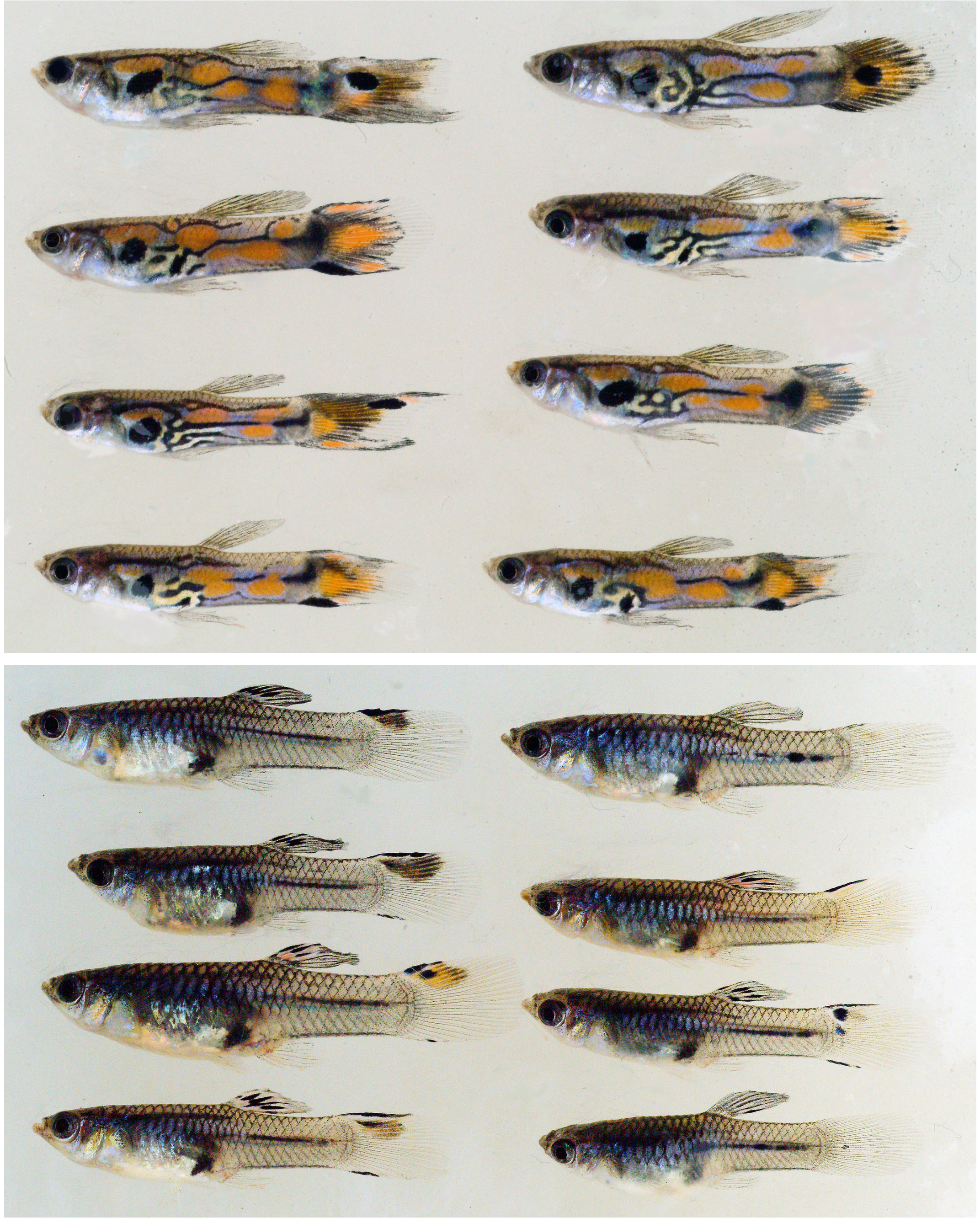

Supplementary Figure S6: Males (upper) and testosterone treated females (lower) of *P. reticulata*, strain IstW

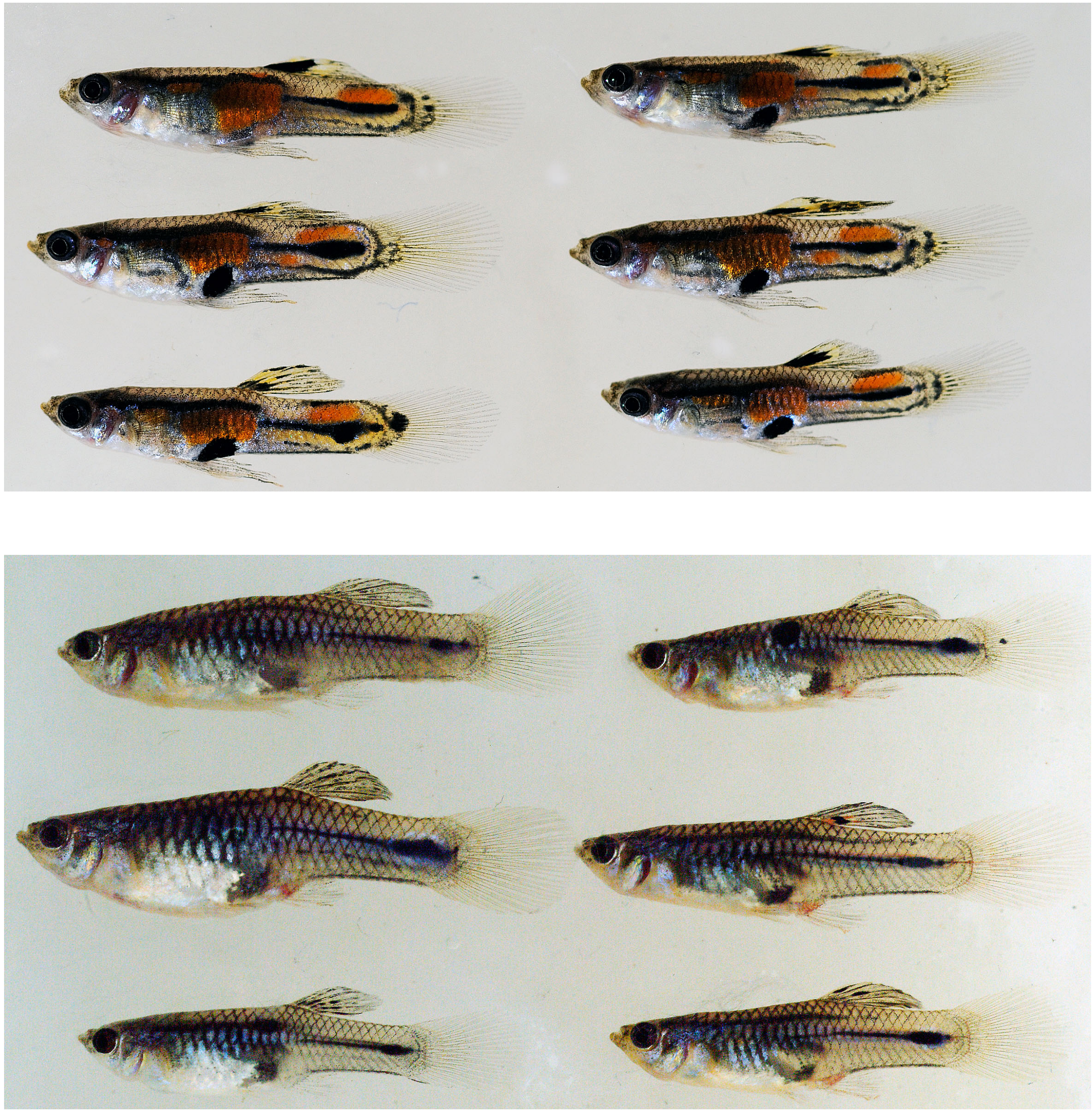

Supplementary Figure S7: Males (upper) and testosterone treated females (lower) of *P. reticulata*, strain ma-ze.

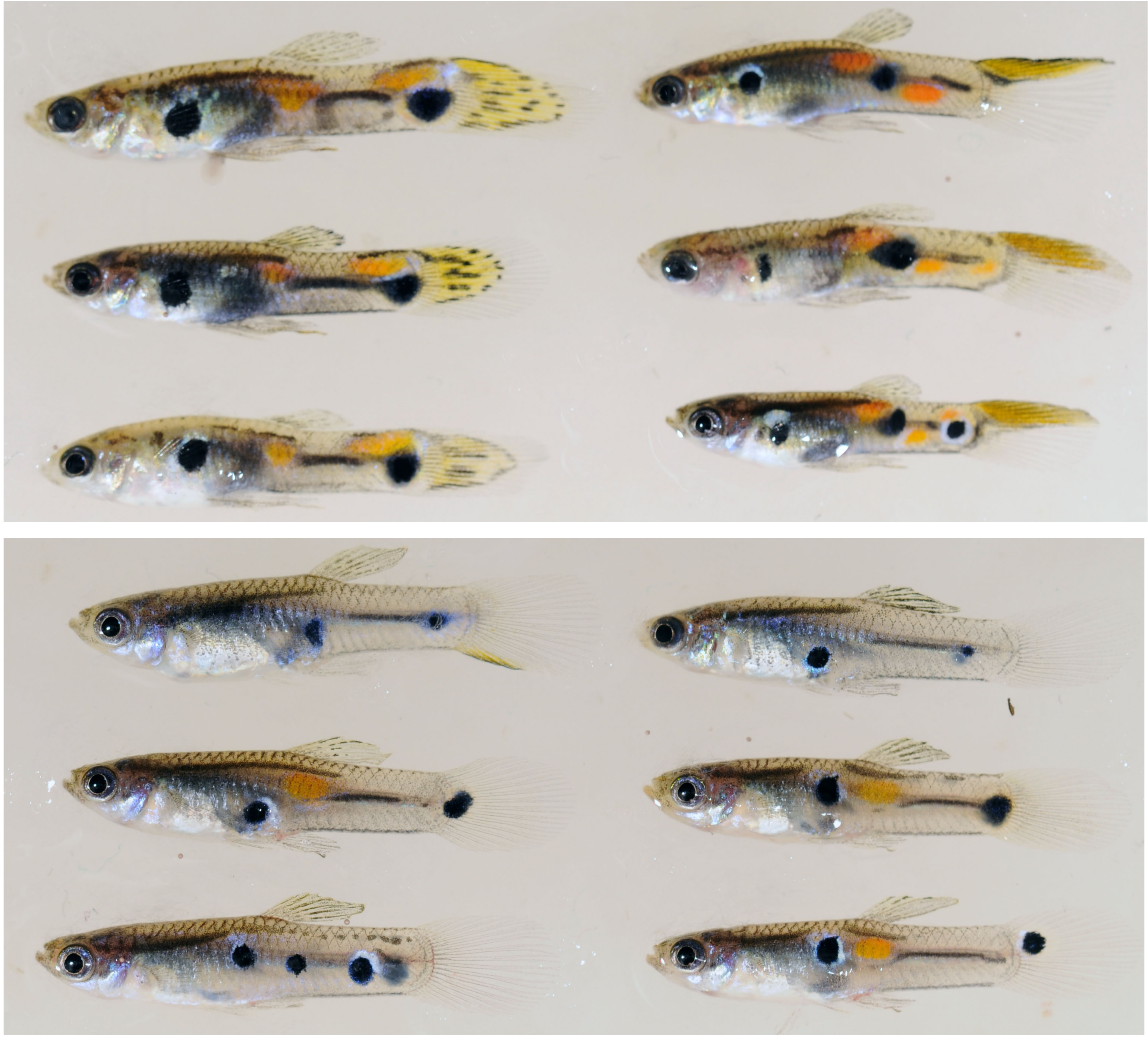

Supplementary Figure S8: Males (upper) and testosterone treated females (lower) of *P. reticulata*, strain HR.

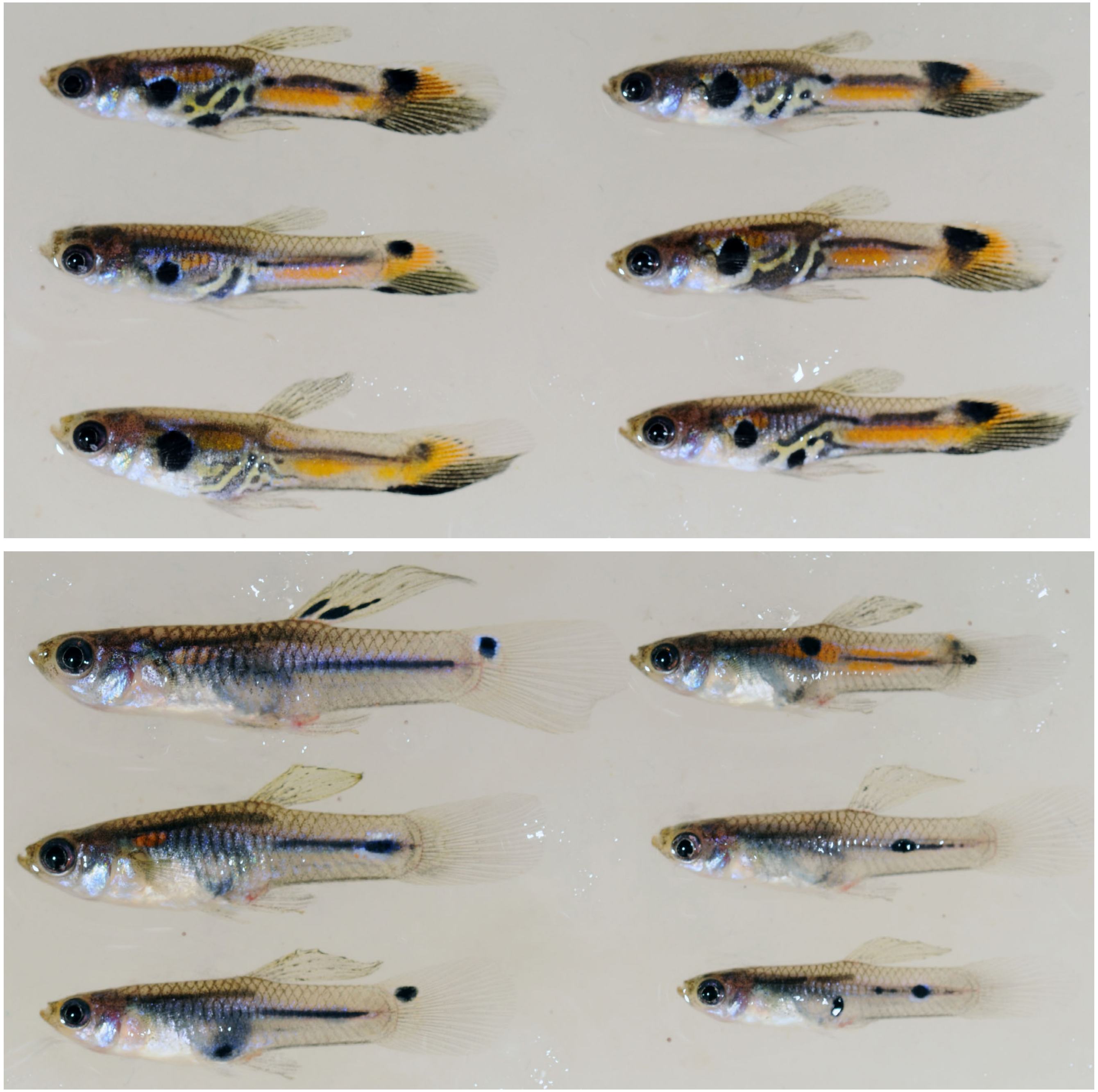

Supplementary Figure S9: Males (upper) and testosterone treated females (lower) of *P. reticulata*, strain HR.

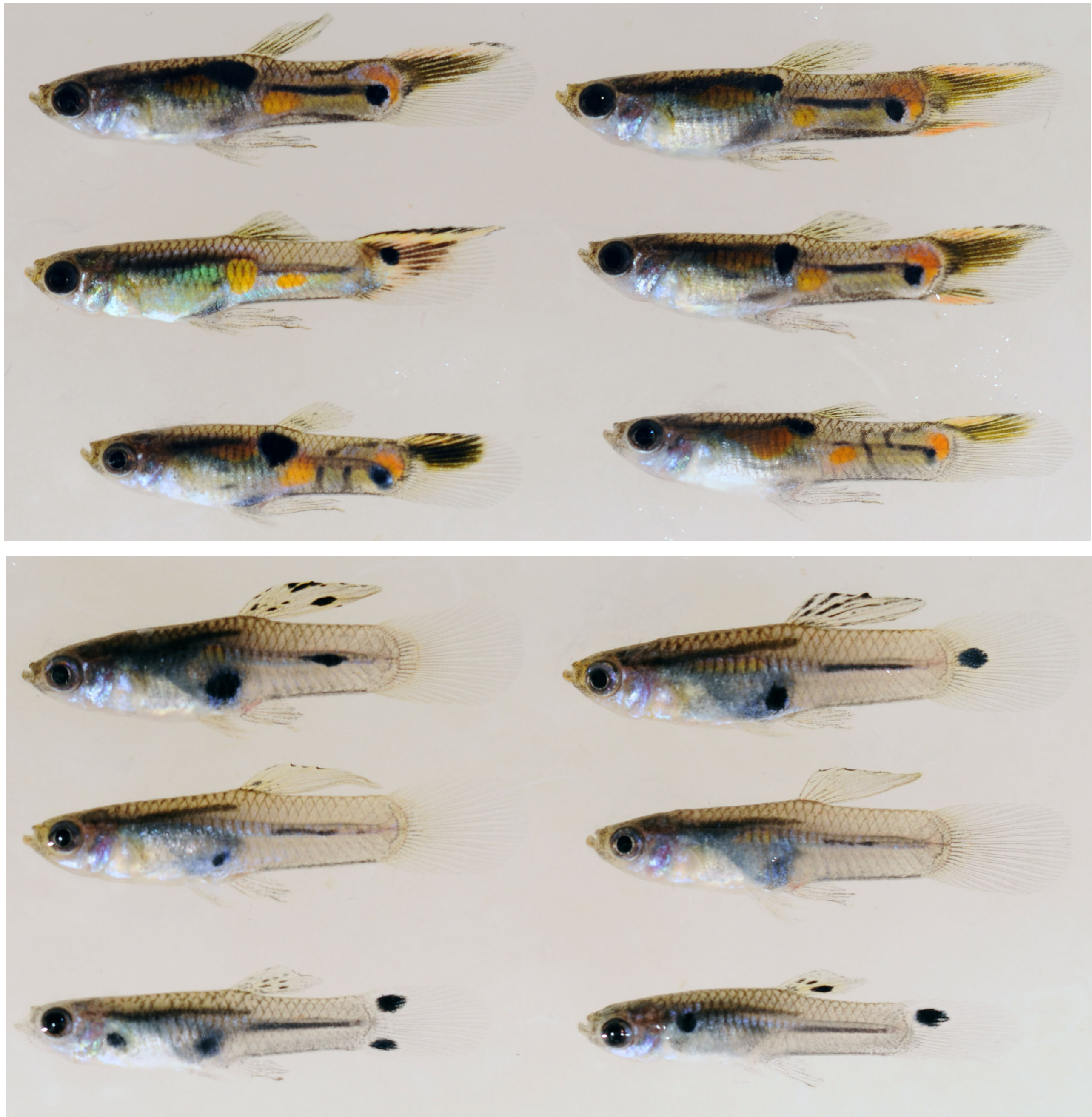

Supplementary Figure S10: Males (upper) and testosterone treated females (lower) of *P. obscura*, strain OR.

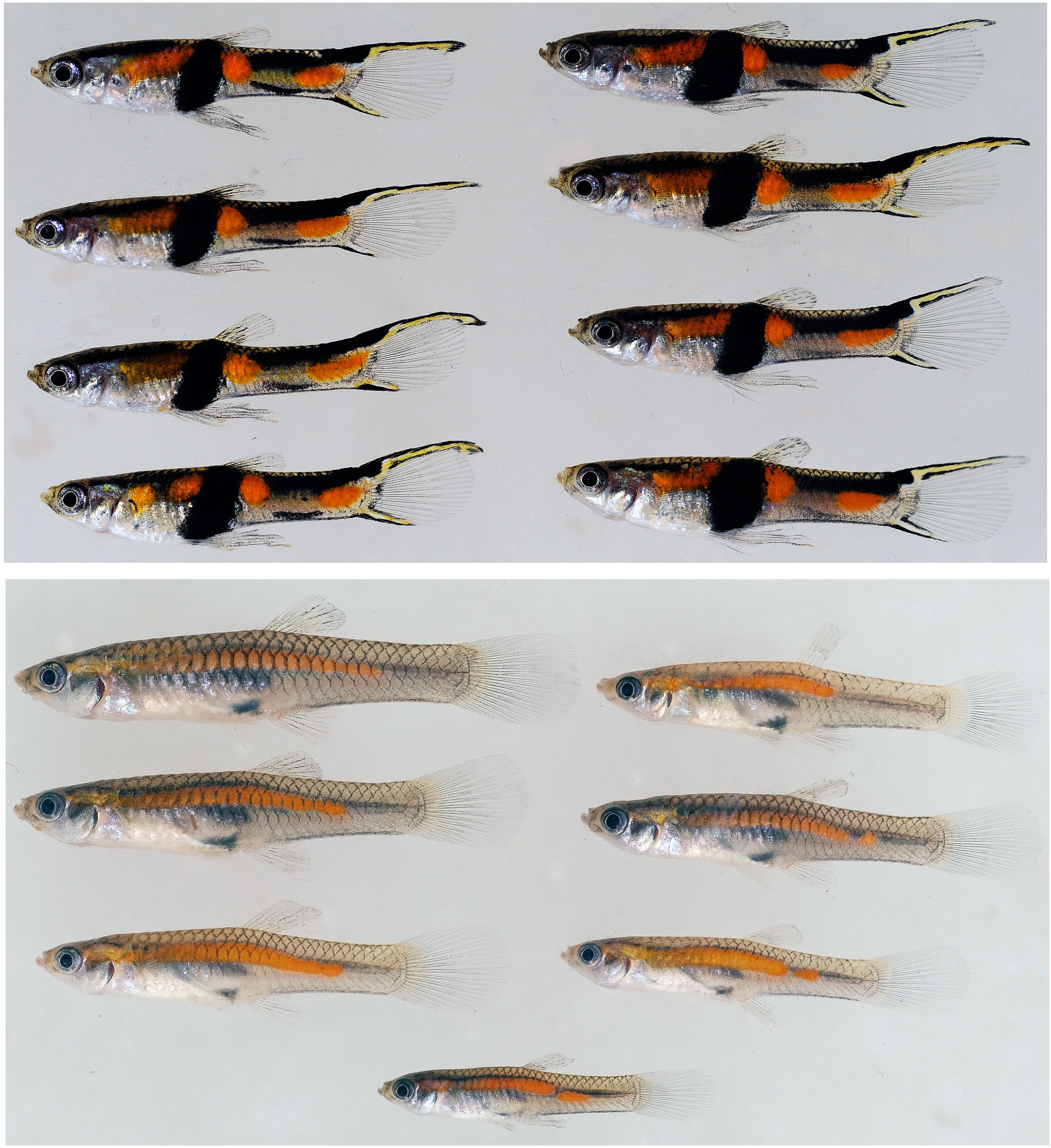

Supplementary Figure S11: Males (upper) and testosterone treated females (lower) of *P. wingei*, strain Ca.

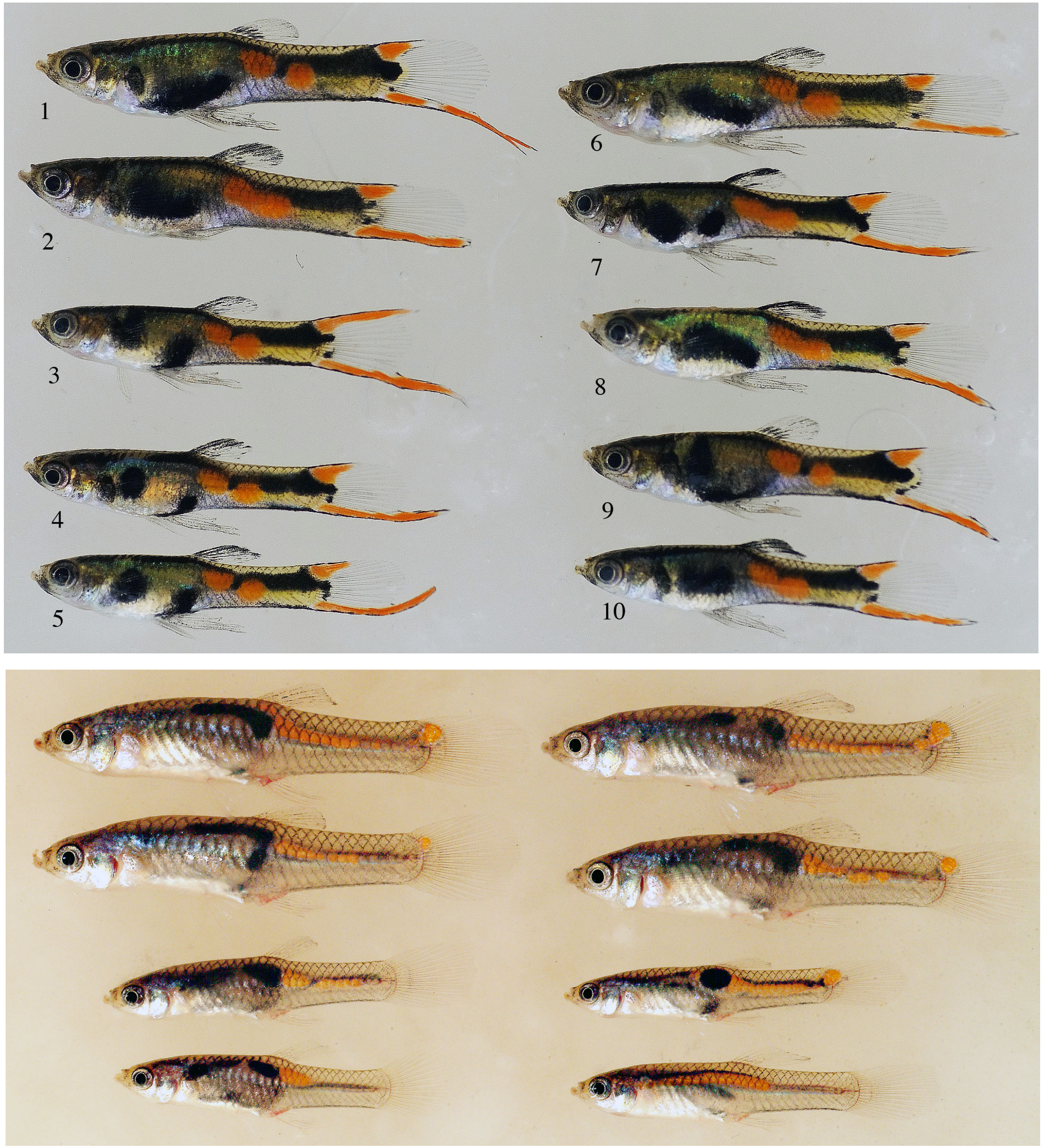

Supplementary Figure S12: Males (upper) and testosterone treated females (lower) of *P. wingei*, strain LP, laboratory line 3521

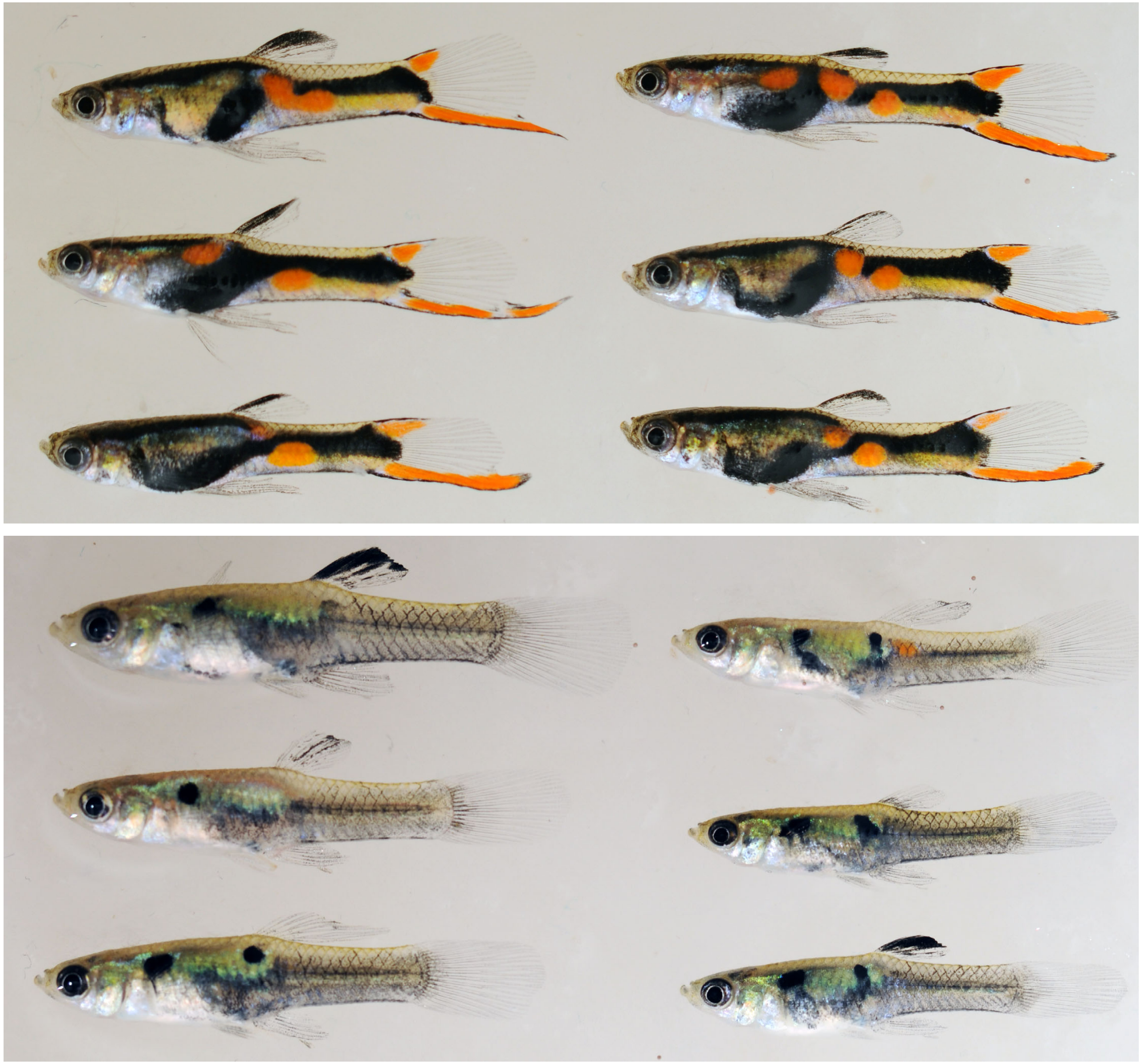

Supplementary Figure S13: Males (upper) and testosterone treated females (lower) of *P. wingei*, strain LP, laboratory line 3521b

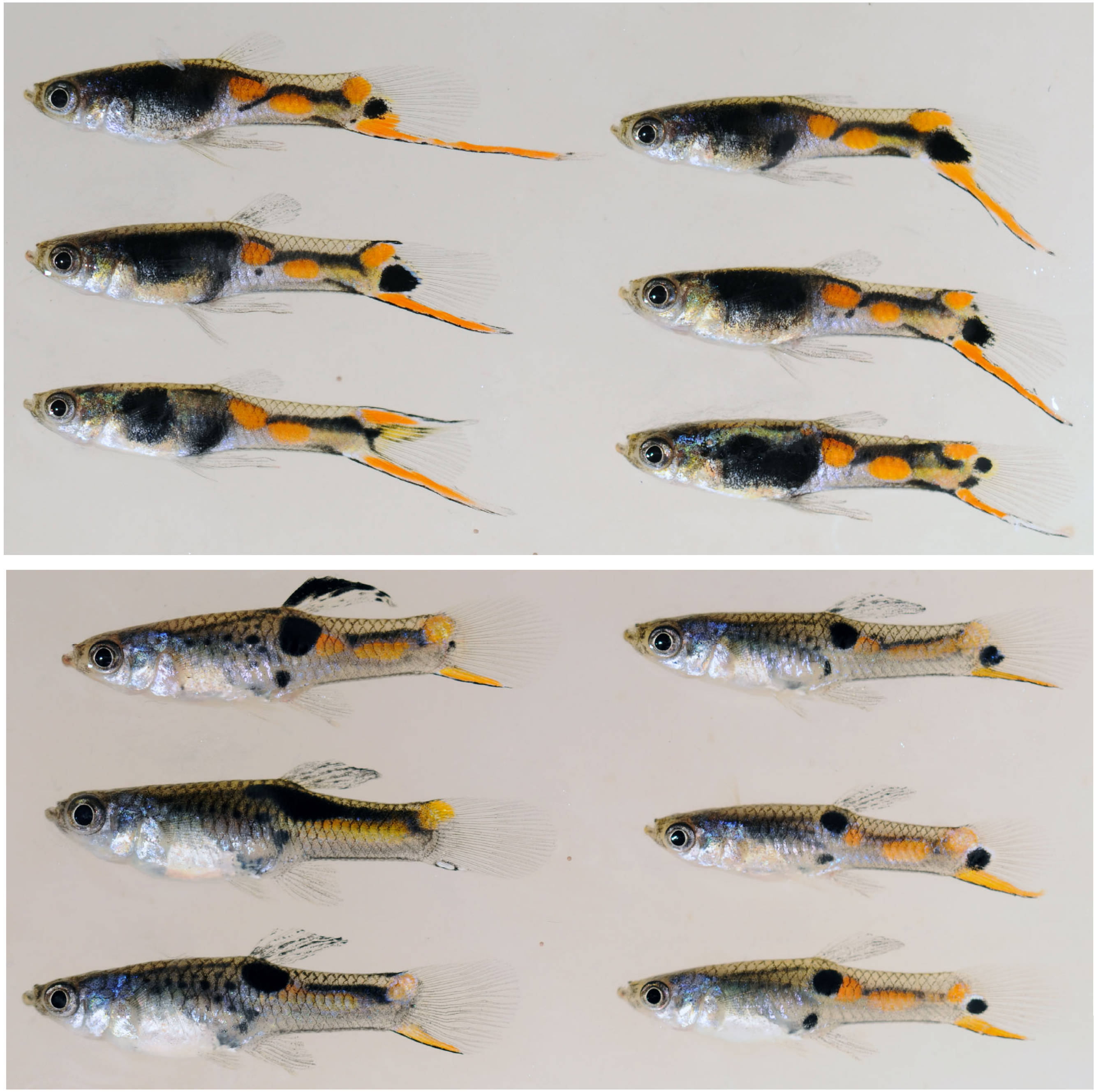

Supplementary Figure S14: Males (upper) and testosterone treated females (lower) of *P. wingei*, strain LP, laboratory line 5898

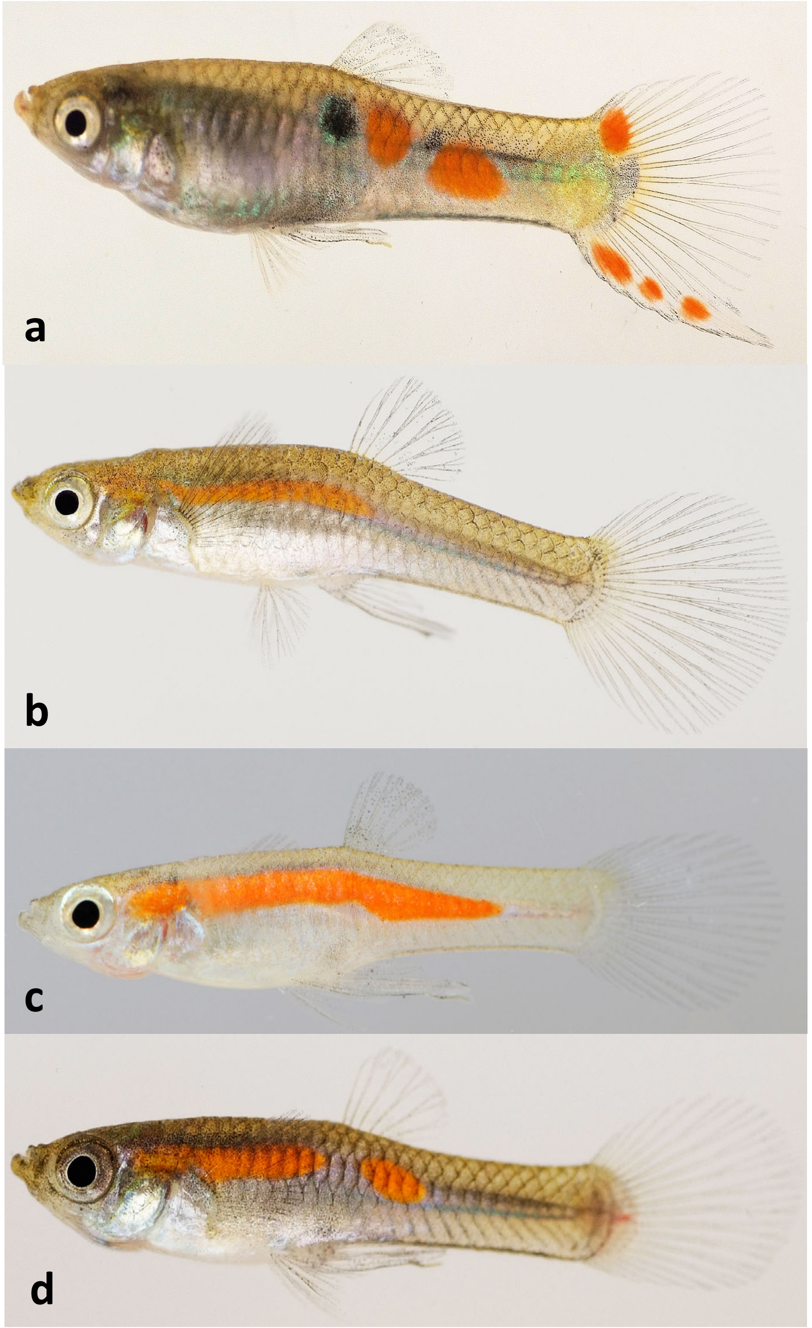

Supplementary Figure S15: XX males of *P. wingei.* (a) strain LP (b-d) strain Ca.

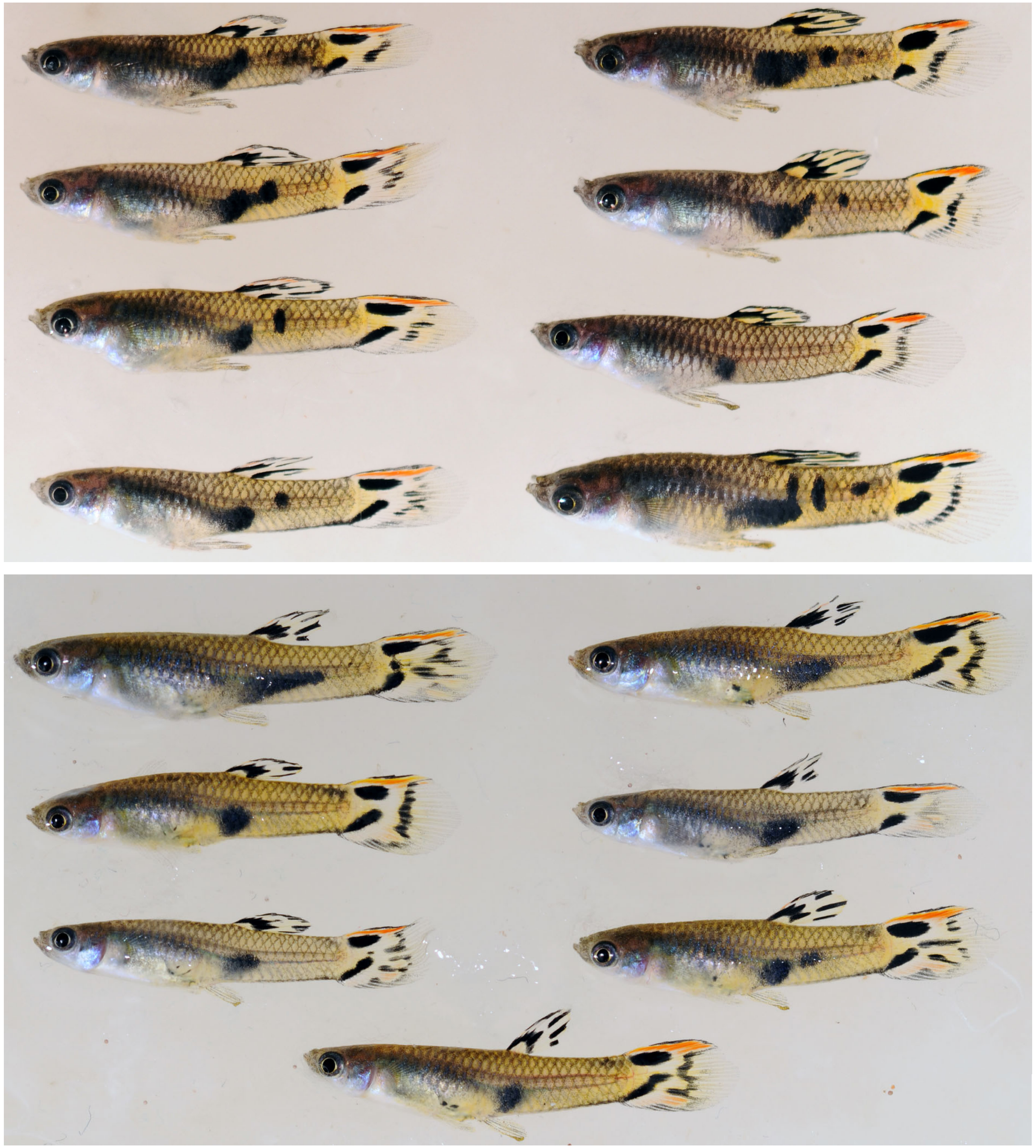

Supplementary Figure S16: Males (upper) and testosterone treated females (lower) of *M. picta*, strain TR.

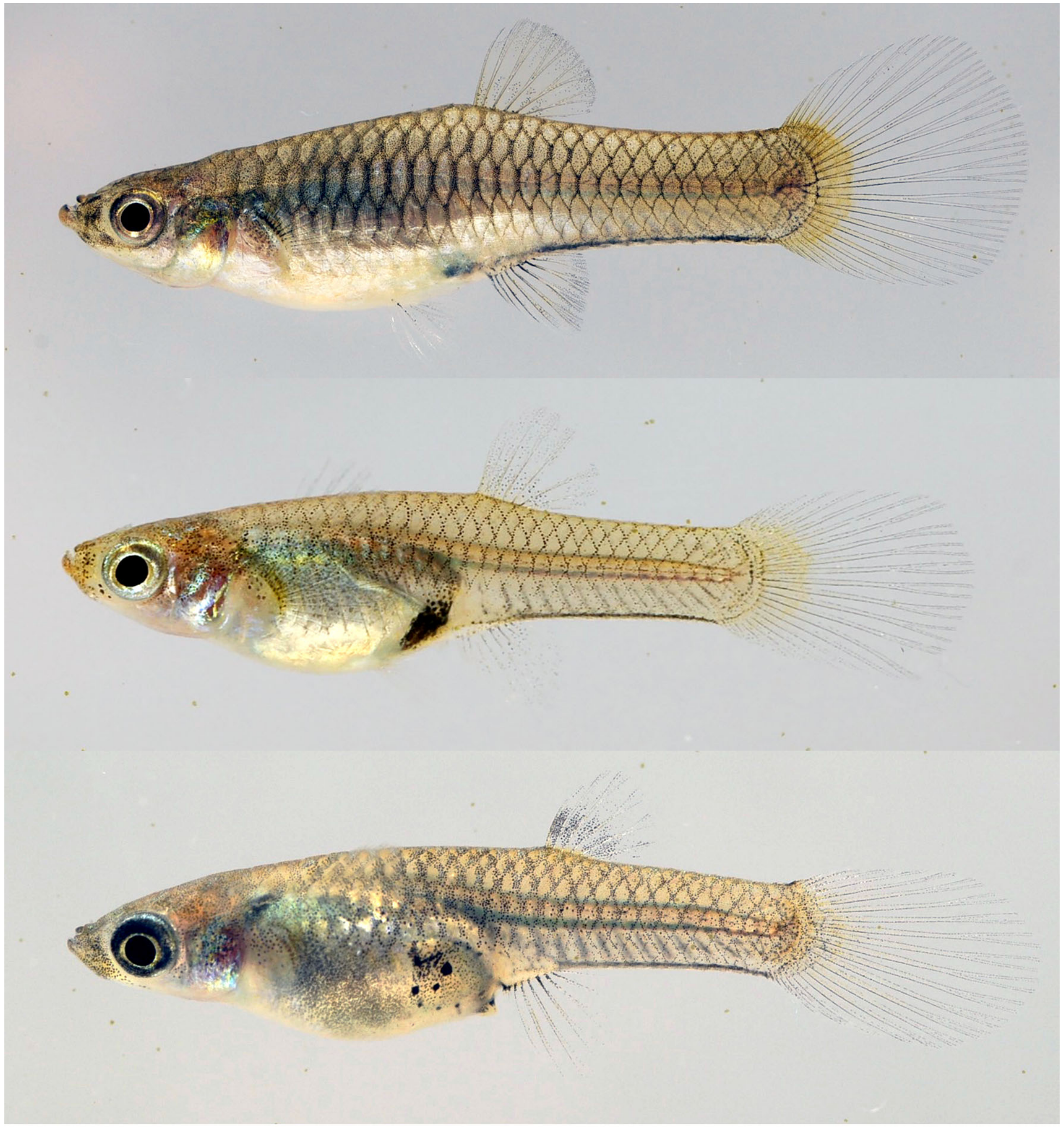

Supplementary Figure S17: Untreated females of *P. wingei* (upper), *P. reticulata* (middle), *M. picta* (lower). Untreated females of *P. obscura* are phenotypically undistinguishable from females of *P. reticulata*.
